# Weighted technical and scale efficiency of Greek public general hospitals grouped by bed capacity: a DEA analysis (2019–2023)

**DOI:** 10.1108/JHOM-03-2026-0326

**Published:** 2026-11-10

**Authors:** Evangelos Gkoutzias

**Affiliations:** Department of Accounting and Finance, University of Ioannina, Preveza, Greece

**Keywords:** Data envelopment analysis, Technical efficiency, Scale efficiency, Public hospitals, Health system performance, Greece

## Abstract

**Purpose:**

This study examines the weighted technical efficiency and scale efficiency of Greek public general hospitals within the National Health System during the period 2019–2023, grouping hospitals by capacity size (small ≤ 200 beds, medium 201–400 beds, large ≥ 401 beds). The analysis investigates efficiency differentials across hospital size categories and the evolution of operational performance before and after the COVID-19 pandemic in a context of sustained fiscal pressure on public health systems.

**Design/methodology/approach:**

Efficiency is assessed using Data Envelopment Analysis (DEA), applying Constant Returns to Scale (CRS) and Variable Returns to Scale (VRS) models. Indicators of technical efficiency, pure technical efficiency and scale efficiency (SE = CRS/VRS) are estimated using the MaxDEA Lite software. Weighted annual efficiency scores are calculated by hospital size group, enabling consistent intertemporal comparisons and a clearer interpretation of structural performance differences across hospital categories.

**Findings:**

The results reveal variations in technical and scale efficiency across hospital size groups and differences in operational performance during the study period. Overall, efficiency shows a gradual post-pandemic improvement, indicating enhanced managerial adaptability and more effective resource utilisation, although structural differences between hospital size categories remain evident.

**Research limitations/implications:**

The study is subject to certain limitations. First, the analysis relies on administrative input–output data and does not incorporate indicators of quality of care, case severity, or clinical outcomes. Second, the results may be influenced by the exceptional conditions of the COVID-19 pandemic, which affected hospital operations and demand patterns. Third, spatial and demographic factors are not explicitly captured in the model, potentially affecting efficiency estimates. Future research could incorporate quality indicators, extend the analysis to other contexts, and apply complementary methodologies for a more comprehensive evaluation.

**Practical implications:**

The findings provide actionable insights for policymakers and hospital administrators by highlighting the need for differentiated strategies based on hospital size and scale efficiency. Large hospitals operating under decreasing returns to scale may benefit from service reorganization and improved internal coordination, while smaller and medium-sized hospitals require targeted investments in infrastructure and human resources. The results also support the adoption of performance monitoring systems, benchmarking practices, and digital health technologies to enhance efficiency, transparency, and long-term sustainability within the Greek public health system.

**Social implications:**

The findings highlight disparities in efficiency across hospital size groups, particularly affecting smaller and remote hospitals, which may affect equitable access to healthcare services. Strengthening these units through targeted support and collaborative arrangements can help reduce regional inequalities and improve service availability. Enhancing operational efficiency and resource allocation contributes to better quality of care and supports the long-term sustainability of the public health system.

**Originality/value:**

The study provides empirical evidence on hospital efficiency in Greece and demonstrates the usefulness of weighted DEA for analysing heterogeneous hospital systems. The findings support targeted resource allocation, strengthened collaboration among hospital units and the development of benchmarking mechanisms to improve operational efficiency and long-term system sustainability.

## 1. Introduction

Following a prolonged economic crisis and the COVID-19 pandemic, the Greek public healthcare system experienced substantial financial and operational pressures that affected its sustainability and resilience (Athanasakis *et al.*, 2022; Economou, 2024; Kyriopoulos *et al.*, 2025). Public hospitals faced increased demand for services, staffing shortages, resource constraints, and significant organisational challenges during both the pandemic and post-pandemic periods (Economou, 2024). At the same time, the system remained heavily hospital-oriented, raising concerns regarding the efficient allocation and utilisation of healthcare resources. These conditions highlighted the importance of evaluating hospital performance and technical efficiency in order to support evidence-based policymaking, improve operational management, and strengthen the long-term sustainability of the Greek National Health System. Consequently, improving hospital efficiency has become a central priority for health policymakers worldwide, particularly in publicly funded healthcare systems operating under fiscal constraints. Within this framework, the measurement of technical efficiency constitutes a core component of health system performance evaluation under conditions of resource scarcity. Therefore, assessing the technical and scale efficiency of Greek public general hospitals is essential for understanding how hospital size and resource allocation influenced operational performance during and after the COVID-19 pandemic (Athanasakis *et al.*, 2022). Among the available efficiency assessment approaches, Data Envelopment Analysis (DEA) has been widely applied in healthcare research due to its ability to evaluate the relative efficiency of homogeneous Decision Making Units (DMUs) using multiple inputs and outputs without requiring a predefined production function (Charnes *et al*., 1978; Banker *et al*., 1984; Cooper *et al*., 2007). DEA constructs an empirical production frontier based on observed best-practice units. Unlike parametric approaches, it does not require specification of a functional form for the production technology. Efficiency is measured relative to the best-performing units within the sample, with efficiency scores ranging between 0 and 1, where unity indicates that a DMU lies on the efficient frontier.

Internationally, DEA has been widely applied in healthcare, particularly in hospital settings, to assess efficiency and address a range of managerial and policy-related questions (Kohl *et al.*, 2019). Higher efficiency, as calculated by DΕΑ, indicates a more favourable relationship between inputs and outputs. For example, in an output-oriented framework, efficiency may improve when a hospital maintains or increases its service outputs without a proportional increase in the resources and inputs used. In the input-oriented framework adopted in this study, higher efficiency indicates operation closer to the best-practice frontier. In other words, with outputs held constant, efficiency improves when the corresponding inputs are reduced (Coelli *et al.*, 2005). The resources used as inputs are physicians, nurses, beds, operating expenditures, and pharmaceutical expenditures, while the services produced as outpus are inpatient discharges, patient days, surgical operations, emergency, outpatient, and day-care visits, and training physicians. Its use extends across different healthcare systems. At the health-system level, for example, Lee (2016) applied DEA to compare the efficiency of healthcare systems across 28 countries, demonstrating its use for cross-country performance assessment and the evaluation of resource utilization. Beyond its methodological role in efficiency measurement, DEA can also provide practically relevant information for healthcare decision-makers. In particular, DEA can support benchmarking by allowing organizations to compare their performance with better-performing units and identify areas in which efficiency improvements may be required (Alfiero *et al.*, 2021).

### 1.1 CCR model (CRS specification)

Under the assumption of Constant Returns to Scale (CRS), proportional input changes lead to proportional output changes. For *n* DMUs using m inputs to produce s outputs, the input-oriented CCR model for DMU_0_ is:


$$\min_{\theta ,\lambda \;}\theta$$

s.t.

$$\Sigma_{j=1}^{n}\;\lambda_{j}x_{ij}\leq \theta \;x_{i,0},i=1,..,m$$

$$\Sigma_{j=1}^{n}\;\lambda_{j}y_{rj}\geq y_{r,0},r=1,\ldots ,s$$

$$\lambda_{j}\geq 0,j=1,\ldots ,n$$

The model determines the maximum feasible proportional contraction of inputs while maintaining observed output levels. In this formulation, θ denotes the technical efficiency score of DMU_0_, and λ_j_ are intensity variables defining the reference benchmark. A value θ = 1 indicates technical efficiency, whereas θ < 1 indicates input inefficiency.

### 1.2 BCC model (VRS specification)

The BCC model introduces the convexity constraint:


$${\Sigma_{j=}^{n}}_{1}\;\lambda_{j}=1,$$

This restriction allows for variable returns to scale and yields a measure of pure technical efficiency, disentangling managerial inefficiency from scale inefficiency (Banker *et al.*, 1984).

Under CRS, efficiency scores capture overall technical efficiency by conflating pure technical inefficiency with scale effects. By contrast, the VRS specification relaxes the proportionality assumption and enables comparisons among units of similar size, providing estimates of pure technical efficiency free from scale effects (Cooper *et al.*, 2007). In addition, the VRS framework permits classification of units into increasing, constant, or decreasing returns-to-scale regimes (Banker *et al.*, 1984; Ozcan, 2014).

### 1.3 Orientation and scale efficiency

DEA models may be specified as input-oriented (input contraction) or output-oriented (output expansion) (Cooper *et al.*, 2007). Efficiency is inherently relative to the empirically constructed frontier.

Scale efficiency (SE) is defined as the ratio of overall technical efficiency (CRS) to pure technical efficiency (VRS):


$$SE=\frac{\theta_{CRS}}{\theta_{VRS}}$$

This ratio indicates whether a DMU operates at its most productive scale size; values below unity indicate scale inefficiency.

An input-oriented specification was adopted, reflecting the greater managerial control over resource utilization than over healthcare service demand in publicly funded hospital systems (Jacobs *et al*., 2006; Worthington, 2004).

### 1.4 Methodological considerations

Despite its advantages, DEA does not explicitly account for random noise and remains sensitive to model specification and variable selection. Accordingly, efficiency scores are interpreted as relative and exploratory performance indicators rather than absolute or normative measures of effectiveness (Simar and Wilson, 2007). Mean efficiency estimates should be interpreted primarily as indicative system-level patterns, given the heterogeneity across hospital units.

Beyond conventional DEA models, the efficiency literature has proposed complementary approaches, including bootstrap DEA, stochastic frontier analysis (SFA), and Malmquist productivity indices, to address limitations related to the deterministic nature of DEA and sensitivity to statistical noise and temporal dynamics (Aigner *et al.*, 1977; Simar and Wilson, 2000; Tziogkidis, 2012). Nevertheless, conventional DEA remains widely applied in hospital efficiency studies due to its flexibility in handling multiple inputs and outputs without requiring predefined production functions.

DEA also serves as a benchmarking tool by identifying reference units and quantitative improvement targets, thereby supporting evidence-based resource allocation in public health systems (Hollingsworth, 2008; Ozcan, 2014). A key strength of the method lies in its ability to distinguish pure technical inefficiency from scale inefficiency, a distinction particularly relevant in heterogeneous hospital systems where ignoring scale effects may lead to misleading policy inferences.

### 1.5 Orientation selection

An input-oriented DEA specification was adopted because hospital administrators in publicly funded healthcare systems generally exercise greater control over resource utilisation than over healthcare demand and service outputs. This orientation is widely applied in hospital efficiency studies where the objective is to assess the extent to which input resources can be reduced while maintaining observed output levels (Jacobs *et al.*, 2006; Worthington, 2004).

### 1.6 Input and output selection

The selection of input and output variables was based on established hospital efficiency literature, while also considering the availability and consistency of data within the Greek public healthcare system (Ozcan, 2014; Kohl *et al.*, 2019; Fourlopoulou *et al.*, 2025). The selected inputs represent key human and infrastructural resources used in hospital production processes, whereas the outputs reflect major dimensions of healthcare service delivery and hospital activity. Variable selection aimed to capture operational performance and resource utilisation across heterogeneous hospital units while ensuring consistency with established DEA applications in healthcare settings.

This study contributes to the literature on hospital efficiency by examining the technical efficiency of public general hospitals in Greece during the period 2019–2023 using Data Envelopment Analysis (DEA). By distinguishing between Constant Returns to Scale (CRS) and Variable Returns to Scale (VRS) and analysing scale efficiency across hospital size groups, the study provides insights into structural efficiency differences within the Greek National Health System.

## 2. Literature review

The comprehensive literature review by Emrouznejad and Yang (2018), covering forty years of DEA applications, confirms the continuous expansion of the method's fields of application, particularly in the health sector. As highlighted by Cooper *et al*. (2011) and Ozcan (2014), DEA constitutes a reliable tool for assessing technical efficiency and identifying the Most Productive Scale Size (MPSS) through the joint analysis of CRS and VRS efficiency measures.

Efficiency assessment using DEA has also been applied across different hospital ownership settings. Guerrini *et al.* (2018), for example, employed DEA to assess efficiency in both public and private hospitals within the Italian regional health system, examining the role of ownership in hospital performance. More broadly, Kruse *et al.* (2018) reviewed efficiency evidence across public, private for-profit, and private not-for-profit hospitals in European healthcare systems. In the private sector, DEA-based performance assessment has been used to support hospital management in making informed choices aimed at financial sustainability and sustainable development (Kourtis *et al.*, 2021). In the public sector, DEA-based approaches can support government resource-allocation and planning decisions by providing an efficiency-based framework for the centralized allocation of healthcare resources (Du *et al.*, 2025).

International evidence reports comparable efficiency patterns in hospital sectors across Middle Eastern countries. In Saudi Arabia, Alatawi *et al.* (2019) evaluated the efficiency of public hospitals using DEA under both CCR (CRS) and BCC (VRS) models. Their findings indicated relatively high levels of technical efficiency, with a mean score of approximately 0.76, alongside substantial variation in optimal operating scale. More than half of Saudi hospitals were found to operate under decreasing returns to scale, signaling non-optimal scale conditions and the need for resource reallocation.

The cross-country study by Varabyova and Schreyögg (2013), analysing hospital data from 25 OECD countries over the period 2000–2009, documented significant variation in technical efficiency levels across countries. Despite these differences, most estimates clustered within relatively high efficiency ranges, typically between 0.80 and 0.90.

Within the European context, recent DEA applications consistently report high levels of technical efficiency in public hospitals. In Portugal, the study by Afonso *et al.* (2024) does not provide explicit numerical efficiency values; however, visual inspection of the reported figures suggests average VRS efficiency scores ranging approximately between 0.83 and 0.89. In France, Hadji and Degoulet (2023) analyzed twenty university hospitals using input-oriented VRS DEA and reported technical efficiency scores between 0.87 and 0.99 for model M1 and between 0.87 and 1.00 for model M2, although corresponding CRS estimates were not reported. Similarly, for Spain, Tapia and Salvador (2022) documented high average VRS efficiency values ranging from 0.91 to 0.96. Overall, the European literature indicates that public hospitals consistently achieve high levels of technical efficiency under VRS specifications. These findings are aligned with the results of the present study, where efficiency differentials are driven primarily by scale-related and organizational factors rather than by pure technical constraints.

Evidence from developing countries further reinforces the importance of scale and organisational capacity in shaping hospital efficiency. In Namibia, Zere *et al.* (2006) reported average technical efficiency levels below 0.75, indicating considerable scope for improvement, largely attributable to non-optimal operating scale and limited managerial capacity. Similarly, Alhassan *et al.* (2015) found mean technical efficiency of approximately 0.65 in Ghanaian hospitals, with particularly low performance among regional and smaller facilities. These inefficiencies were associated with organizational weaknesses, staffing shortages and constrained access to resources. Overall, the international literature confirms that public hospital efficiency in developing countries is strongly influenced by size, regional disparities and organizational maturity, with the most pronounced efficiency losses arising from non-optimal production scale and insufficient utilization of available technological and managerial resources.

In Greece, Kontodimopoulos *et al.* (2006) evaluated 17 NHS hospital–health centers and reported technical inefficiency levels of 25–27%. Remote island hospitals exhibited lower efficiency, while facilities providing preventive services demonstrated superior performance. Similarly, Flokou (2017), analyzing 73 general NHS hospitals for the year 2009, reported mean technical efficiency of 0.79 under VRS and overall efficiency of 0.67 under CRS, indicating that most hospitals operated below their optimal scale. The consolidation and reorganization of similar units was proposed as a potential efficiency-enhancing strategy. More recently, Mitakos and Mpogiatzidis (2024), examining 38 public hospitals in 2020, reported average technical efficiency scores of 0.83 under VRS (BCC) and 0.65 under CRS (CCR), pointing to substantial efficiency losses attributable to non-optimal scale of operation. Collectively, these findings provide a coherent comparative framework within which the present analysis of Greek public hospitals over the period 2019–2023 is situated.

## 3. Research objectives

In the present DEA framework, hospital efficiency reflects the relationship between the resources used as inputs –physicians, nurses, beds, operating expenditures, and pharmaceutical expenditures–and the services produced as outputs–inpatient discharges, patient days, surgical operations, emergency, outpatient and day-care visits, and training physicians.

To address existing gaps in the literature and to strengthen the evidence-based evaluation of public general hospitals within the Greek National Health System (NHS), the present study pursues the following research objectives:

1. To assess the scale efficiency of Greek public general hospitals by size category over the period 2019–2023 and identify their returns-to-scale (RTS) regimes.
2. To assess technical efficiency under Constant Returns to Scale (CRS) and Variable Returns to Scale (VRS) assumptions and examine efficiency trends across hospital size categories over the pre-pandemic, pandemic, and early post-pandemic phases of 2019–2023.
3. To interpret the findings in relation to international evidence and derive implications for resource use, organizational improvement, and scale optimization in Greek public general hospitals.

## 4. Data and methodology

### 4.1 Sample and period of analysis

The present study examines the technical efficiency of public hospitals within the Greek National Health System (NHS) using Data Envelopment Analysis (DEA), implemented through the MaxDEA Lite software, over the period 2019–2023. The sample comprises 97–98 public general hospitals, reflecting institutional changes during the study period: 97 hospitals were operating in 2019, while from 2020 to 2023 the number increased to 98.

These hospitals provide secondary health care services and cover the entire geographical territory of Greece, ensuring representativeness in both spatial and functional terms. For reasons of homogeneity, General Hospitals-Health Centers (GH-HCs) were also included in the sample, as they constitute official NHS hospital units with registered bed capacity and provision of core secondary care services. GH-HCs exhibit operational characteristics comparable to small general hospitals, particularly in island, mountainous, and border regions, where they often represent the sole hospital infrastructure.

Specialized hospitals-such as pediatric, psychiatric, oncology, and other highly specialized institutions-were excluded from the analysis in order to ensure sample homogeneity and comparability of operational characteristics across units. Only hospitals with complete, reliable, and consistent input and output data were included, as provided by the Statistical Service of the Ministry of Health and the ESY.net information system.

Hospitals were classified into three bed-capacity categories, consistent with prior DEA-based hospital efficiency studies examining scale-related performance differences among public hospitals (Flokou *et al.*, 2017; Fourlopoulou *et al.*, 2025):

1. **Small hospitals:** ≤200 beds
2. **Medium-sized hospitals:** 201–400 beds
3. **Large hospitals:** ≥401 beds

This classification facilitates comparisons among hospitals with similar productive capacity, organizational structure, and operational complexity, while minimizing the influence of exogenous factors unrelated to efficiency performance. Overall, the sample covers more than 90% of the main hospital units providing secondary and tertiary care in Greece, effectively representing the public hospital sector as a whole.

The selection of the 2019–2023 period allows for the assessment of NHS performance before, during, and after the COVID-19 pandemic, including the early post-pandemic phase (2022–2023). This approach enables a dynamic evaluation of efficiency changes and system adaptability under conditions of heightened pressure and policy intervention. The five-year time horizon further supports the identification of structural efficiency trends, while mitigating the influence of short-term fiscal or epidemiological fluctuations.

### 4.2 Input and output variables

The analysis relied exclusively on official administrative data obtained from the ESY.net information system (Hellenic Ministry of Health, 2019–2023), without the application of any normalization or rescaling procedures, in order to preserve the actual operating scale of hospitals. Data processing was limited to aggregation procedures for the construction of total input and output variables, while the original values of the underlying indicators remained unchanged.

Furthermore, the uniform application of the CRS and VRS DEA models across all years of the study period ensured consistency in the evaluation framework and full intertemporal comparability of efficiency results.

The selection of input and output variables was guided by the international literature (Ozcan, 2014; Cooper *et al.*, 2007; Ferreira *et al.*, 2018). These variables, presented in Table 1, capture key dimensions of hospital resource utilization and operational service delivery.

**Table 1:** DEA input and output variables

Inputs	Outputs
Physicians	Inpatients (discharges)
Nurses	Patient days
Beds	Surgical operations
Operating expenditures (€)	Emergency, outpatient, and day-care visits
Pharmaceutical expenditures (€)	Training physicians (FTEs)

**Source(s):** Author’s own work

Financial, human, and operational indicators were incorporated to assess hospital technical efficiency across multiple dimensions of hospital activity. All variables were expressed in absolute values, as the objective was to assess efficiency under the actual operating conditions of hospitals, without normalization relative to national averages or benchmark ratios.

### 4.3 DEA methodology

The DEA implementation followed a three-stage procedure. First, overall technical efficiency was estimated under the assumption of Constant Returns to Scale (CRS) using the CCR model proposed by Charnes *et al*. (1978). Second, pure technical efficiency was estimated under Variable Returns to Scale (VRS) using the BCC model developed by Banker *et al*. (1984). Third, scale efficiency (SE) was computed as the ratio of CRS to VRS efficiency scores (SE = TE_CRS/TE_VRS), while the type of returns to scale-Increasing (IRS), Constant (CRS) or Decreasing (DRS)-was identified, allowing the positioning of each unit relative to the Most Productive Scale Size (MPSS).

Within the DEA framework, efficiency scores obtained under CRS are, by construction, less than or equal to those obtained under VRS, as CRS efficiency incorporates both pure technical inefficiency and scale inefficiency. In contrast, VRS efficiency isolates pure technical efficiency by controlling for scale effects, systematically yielding higher or equal scores relative to CRS. The divergence between CRS and VRS efficiency therefore captures scale efficiency and provides critical insight into whether deviations from the efficiency frontier are attributable to managerial practices or to structural scale mismatches (Banker *et al.*, 1984; Cooper *et al*., 2007; Ozcan, 2014).

An input-oriented DEA model was adopted, reflecting the institutional context of the Greek NHS, where hospital management exercises greater control over resource utilization than over service demand. This orientation is widely applied in hospital efficiency studies and is well established in the relevant literature (Worthington, 2004; Hollingsworth, 2008).

DEA computations were performed using MaxDEA Lite on an annual basis for each hospital. Results were subsequently grouped into three bed-capacity categories (small, medium and large hospitals), and weighted mean efficiency scores were calculated using the number of beds as weights. The analysis included both within-group comparisons and intertemporal assessment over the period 2019–2023, enabling the identification of structural efficiency trends and changes associated with the COVID-19 pandemic and administrative reforms within the NHS.

To ensure that intertemporal comparisons were not confounded by changes in sample composition or production conditions, DEA was implemented as a year-specific analysis for each year of the period 2019–2023, using a consistent model specification in terms of inputs, outputs and orientation. Efficiency scores for general hospitals were estimated relative to the national efficiency frontier of the corresponding year, defined by the full set of 122 NHS public hospitals with available data during each period, while the present study focuses exclusively on the subset of 97–98 general hospitals.

The use of a common reference frontier per year is methodologically crucial, as it ensures that all Decision-Making Units (DMUs) are evaluated within a shared production environment, avoiding fragmentation into local frontiers that would undermine intertemporal and cross-sectional comparability. As emphasized in the literature, evaluation against a common frontier is a prerequisite for reliable relative efficiency estimation and meaningful system-level benchmarking (Cooper *et al*., 2011; Ozcan, 2014).

Importantly, no separate DEA was conducted exclusively for the subset of general hospitals, thereby avoiding artificial inflation of efficiency scores arising from increased sample homogeneity (sample selection bias). Each general hospital was therefore assessed relative to the entire public hospital system, strengthening the external validity of the findings and accurately reflecting its relative position within the national performance landscape.

### 4.4 Returns to scale (RTS) identification

Within the framework of Data Envelopment Analysis (DEA), returns to scale are classified as Increasing Returns to Scale (IRS), Constant Returns to Scale (CRS), or Decreasing Returns to Scale (DRS). This classification constitutes a structural property of the production technology and is conceptually independent of technical efficiency scores.

According to DEA theory, the identification of returns to scale is conducted exclusively within the Variable Returns to Scale (VRS) framework, as formalized in the Banker–Charnes–Cooper (BCC) model. The classification is based on the sign of the intercept term u0u_0u0​ (or equivalently −u0-u_0−u0​) in the dual formulation of the BCC model (Banker *et al*., 1984). This term reflects the relative position of each decision-making unit with respect to the Most Productive Scale Size (MPSS).

In the present study, the returns-to-scale (RTS) regime was identified using the RTS Constant (−u0-u_0−u0​) derived from the dual BCC model, as computed by the MaxDEA Lite software. In the results tables, returns to scale are reported symbolically as IRTS, CRTS, and DRTS, solely as descriptive indicators of the scale regime implied by the VRS/BCC framework and not as distinct DEA model specifications.

For operational RTS classification purposes, a ±0.02 tolerance threshold around zero was applied to avoid classifications driven by marginal numerical deviations arising from the numerical solution of linear programming problems. RTS identification remained based on the standard BCC/VRS framework (Banker *et al*., 1984). The tolerance criterion was introduced solely to improve numerical stability in the RTS assignment process, consistent with numerical stability and sensitivity considerations discussed in DEA literature (Seiford and Zhu, 1999; Cooper *et al*., 2007).

1. ∣RTS∣≤0.02 → Constant Returns to Scale (CRTS)
2. RTS<−0.02 → Decreasing Returns to Scale (DRTS)
3. RTS> 0.02 → Increasing Returns to Scale (IRTS)

A clear distinction between technical efficiency scores and returns to scale is critical for the correct interpretation of DEA results. Technical efficiency scores under Constant Returns to Scale (CRS efficiency scores) are, by definition, less than or equal to those obtained under Variable Returns to Scale (VRS efficiency scores), since the CRS model is a restricted special case of the VRS model. The ratio of these two scores defines Scale Efficiency (SE = TE_CRS/TE_VRS), where CRS and VRS refer to technical efficiency estimates rather than to scale regimes. Scale efficiency therefore captures the portion of overall inefficiency attributable exclusively to non-optimal operating scale.

By contrast, returns to scale (RTS) represent an inherent characteristic of the production technology and do not result from a comparison between CRS and VRS efficiency scores. Instead, they reflect the direction of output change in response to proportional increases or decreases in all inputs. Under Increasing Returns to Scale (IRTS), a unit operates below the MPSS, as proportional input expansion leads to a more-than-proportional increase in outputs. Under Decreasing Returns to Scale (DRTS), the unit operates beyond the MPSS, while Constant Returns to Scale (CRTS) indicate operation close to the optimal production scale.

### 4.5 Methodological limitations

The present methodological approach is subject to certain limitations that are inherent to classical applications of Data Envelopment Analysis (DEA) and should be taken into account when interpreting the results.

First, DEA was applied as a pure technical efficiency model, without explicit adjustment for quality differences or case-mix variation. The absence of adjustment mechanisms such as Diagnosis-Related Groups (DRGs) or case-severity indices may affect the precision of efficiency estimates, particularly for hospitals characterized by higher levels of clinical complexity.

Second, the analysis relies on a conventional deterministic DEA framework without statistical correction. Bootstrap DEA techniques were not employed to estimate confidence intervals or to assess the robustness of efficiency scores, thereby limiting the scope for statistical inference.

Third, a two-stage analytical approach was not adopted. Such an approach would allow for the examination of exogenous factors potentially affecting efficiency-such as geographical location, staffing mix, or organizational characteristics—through second-stage regression models (e.g. Tobit or fractional regression).

Finally, the analysis relies on administrative ESY.net data, which despite institutional validation, do not capture qualitative dimensions of hospital performance, such as management quality, workflow efficiency, or digital maturity.

## 5. Results

The DEA analysis reveals substantial differences in both technical efficiency and scale efficiency among Greek general hospitals over the five-year period 2019–2023. The presentation of results is organized at four levels:

1. scale efficiency (SE), which indicates how closely hospitals operate to their most productive scale;
2. weighted technical efficiency under CRS assumptions, reflecting overall technical efficiency;
3. weighted technical efficiency under VRS assumptions, reflecting pure technical efficiency independently of scale effects; and
4. the distribution of returns-to-scale (RTS) regimes by hospital size group, indicating whether hospitals exhibit increasing, constant, or decreasing returns to scale.

### 5.1 Scale efficiency by hospital size group

Based on the results reported in Table 2, mean scale efficiency (SE) remains consistently high over the period 2019–2023, ranging approximately from 0.93 to 0.99. Over the full 2019–2023 period, mean SE was 0.946 for small, 0.963 for medium-sized, and 0.981 for large hospitals. The consistently high SE values indicate relatively limited inefficiency attributable to non-optimal operating scale (Coelli *et al.*, 2005; Ozcan, 2014).

**Table 2:** Mean scale efficiency by hospital size group

Year	Small (≤200)	Medium (201–400)	Large (≥401)
2019	0.945	0.956	0.985
2020	0.945	0.949	0.985
2021	0.963	0.973	0.974
2022	0.931	0.963	0.979
2023	0.944	0.971	0.982
2019–2023	0.946	0.963	0.981

**Note(s):** Values in Table 2 represent the mean of individual hospital scale efficiency scores (SE = TE_CRS/TE_VRS). Consequently, they may differ slightly from the ratio of the weighted mean CRS and VRS scores reported in Tables 3 and 4. The 2019–2023 values represent the mean of all individual hospital-year SE scores over the full study period

**Source(s):** ESY.net (2019–2023), Author’s own work

Large hospitals (≥401 beds) consistently record the highest scale efficiency, with an overall mean SE of 0.981 for 2019–2023 and annual values ranging from 0.974 to 0.985, suggesting operation very close to the Most Productive Scale Size (MPSS). This finding may reflect the ability of larger hospitals to exploit economies of scale and utilize fixed infrastructural and administrative resources more efficiently, as suggested in the hospital efficiency literature (Giancotti *et al.*, 2017). Consistency with the CRS and VRS results indicates strong pure technical and scale efficiency among large hospitals.

Medium-sized hospitals (201–400 beds) maintain high scale efficiency throughout the study period, with an overall mean SE of 0.963 for 2019–2023 and annual values ranging from 0.949 to 0.973. This pattern suggests relatively limited scale-related inefficiencies and may reflect greater flexibility in adjusting operating capacity compared with smaller units.

In contrast, small hospitals (≤200 beds) consistently exhibit lower scale efficiency than the other size groups, with an overall mean SE of 0.946 for 2019–2023 and annual values ranging from 0.931 to 0.963. This indicates greater scale-related inefficiency among smaller hospital units and may reflect persistent structural constraints in achieving economies of scale. These results support the view that, even when pure technical efficiency is relatively satisfactory, operating scale remains an important determinant of overall efficiency for smaller hospital units (Hollingsworth, 2008; Ozcan, 2014).

Overall, the SE analysis complements the CRS and VRS findings by demonstrating that efficiency differentials across hospital size groups are driven to a substantial extent by scale effects. The joint examination of CRS, VRS, and SE provides a coherent and integrated picture of both technical and structural efficiency within the public hospital system.

Taking 2019 as the pre-pandemic benchmark, scale efficiency remained broadly stable across hospital-size categories when compared with overall performance during 2019–2023. For small hospitals, mean SE was virtually unchanged (0.945 in 2019 and 0.946 overall), while medium-sized hospitals showed a slight increase from 0.956 to 0.963. Large hospitals recorded only a marginal decrease, from 0.985 to 0.981.

### 5.2 Weighted CRS technical efficiency by hospital size group

#### 5.2.1 Interpretation

Based on the results reported in Table 3, weighted technical efficiency under Constant Returns to Scale (CRS) ranged overall between approximately 0.82 and 0.96 during the period 2019–2023. Over the full study period, overall bed-weighted CRS efficiency was 0.835 for small, 0.921 for medium-sized and 0.941 for large hospitals.

**Table 3:** Weighted CRS technical efficiency by hospital size group

Year	Small (≤200 beds)	Medium (201–400)	Large (≥401)
2019	0.852	0.904	0.958
2020	0.823	0.905	0.955
2021	0.829	0.928	0.909
2022	0.834	0.932	0.936
2023	0.836	0.930	0.946
2019–2023	0.835	0.921	0.941

**Note(s):** Weighted efficiency scores were calculated using hospital beds as weights. The 2019–2023 values represent the overall bed-weighted efficiency scores calculated from all hospital-year observations over the full study period

**Source(s):** ESY.net (2019–2023), Author’s own work

Large hospitals (≥401 beds) generally exhibit the highest CRS efficiency levels, with an overall bed-weighted efficiency score of 0.941 for 2019–2023 and annual scores ranging from 0.909 to 0.958. This is consistent with theoretical expectations for units operating closer to the optimal scale under the CRS framework. This pattern may reflect economies of scale, more efficient utilization of clinical resources, and greater operational capacity in larger hospital units. Similar associations between hospital size and efficiency performance have been reported in previous DEA studies on Greek public hospitals, which showed that larger hospitals tend to achieve higher operational and economic efficiency levels (Fragkiadakis *et al.*, 2016; Athanassopoulos and Gounaris, 2001). These findings are also broadly consistent with Fourlopoulou *et al.* (2025), who reported that larger hospitals maintained relatively favourable productivity performance compared with smaller units, suggesting that greater resource availability and organizational capacity may contribute to improved operational outcomes. An exception is observed in 2021, when a marked decline in efficiency among large hospitals is recorded (0.909). This deviation coincides with the peak of the COVID-19 pandemic and may reflect the intense operational pressures, resource reallocation, and non-standard production conditions prevailing during that year. Similar pandemic-related productivity deterioration associated with increased healthcare demand and strained resources has been documented in Greek public hospitals by Fourlopoulou *et al.* (2025). Although productivity and technical efficiency represent different performance dimensions, Fourlopoulou *et al.* examined cost productivity changes over time using a Malmquist-based approach, whereas the present study evaluates weighted technical efficiency using DEA under CRS and VRS assumptions. Nevertheless, both studies indicate a deterioration in hospital performance during the peak pandemic period.

Medium-sized hospitals (201–400 beds) display high and relatively stable efficiency throughout the study period, with an overall bed-weighted CRS efficiency of 0.921 for 2019–2023 and annual scores ranging from 0.904 to 0.932, with a mild improvement after 2021. This finding suggests a high degree of operational stability and effective resource utilization among mid-scale units, potentially due to a more balanced relationship between size and organizational structure.

Comparable findings regarding the superior operational performance of medium-sized Greek public hospitals have also been reported in recent Malmquist-based analyses (Fourlopoulou *et al.*, 2025) further reported that medium-sized hospitals ranked first in cost productivity performance during nine of the thirteen years examined and emerged as the overall best-performing hospital category. Fourlopoulou *et al.* (2025) suggested that this superior performance may be explained by the ability of medium-sized hospitals to operate closer to their cost and technological frontiers and to optimise resource utilisation more effectively than smaller and larger units. The convergence of findings obtained through different performance measurement approaches (technical efficiency and productivity analysis) provides additional support for the robustness of the observed hospital-size effects within the Greek public healthcare system. While the present study evaluates weighted technical efficiency using DEA under CRS and VRS assumptions, Fourlopoulou *et al.* assessed cost productivity changes over time and similarly identified medium-sized hospitals as the best-performing category. The consistency of these findings across different methodological approaches strengthens the evidence that medium-sized hospitals may achieve a more balanced utilisation of resources and organisational capacity within the Greek public hospital system.

In contrast, small hospitals (≤200 beds) consistently record lower CRS efficiency scores, with an overall bed-weighted CRS efficiency of 0.835 for 2019–2023 and annual scores ranging from 0.823 to 0.852, with no clear indication of substantial improvement over the five-year period. This pattern suggests that deviations from the efficiency frontier are not solely attributable to temporary external shocks but may also be associated with structural scale constraints and limited managerial flexibility. Comparable evidence has been reported by Fourlopoulou *et al.* (2025), who found that small hospitals consistently exhibited lower cost productivity performance compared with medium-sized hospitals throughout most of the study period.

Overall, the modest upward trend in CRS efficiency indicators after 2021 points to a gradual post-pandemic adjustment and recovery in hospital performance. Beyond these pandemic-related fluctuations, however, the overall 2019–2023 results reveal persistent differences in CRS efficiency across hospital size categories. These differences across the full study period indicate that hospital size and scale-related characteristics remain relevant to overall efficiency performance beyond the year-specific fluctuations observed during the pandemic period. Similar post-pandemic recovery dynamics and inter-hospital variability have been documented in recent DEA-based analyses of Greek public hospitals (Floros *et al.*, 2026). Nevertheless, pronounced efficiency differentials across hospital size categories persist.

A different picture emerges when overall CRS performance is compared with the pre-pandemic benchmark. Relative to 2019, the overall bed-weighted CRS efficiency for 2019–2023 was lower for small hospitals (0.852 vs. 0.835) and large hospitals (0.958 vs. 0.941), whereas medium-sized hospitals showed an increase from 0.904 to 0.921. Thus, the comparison with the pre-pandemic benchmark reveals clear differences in CRS performance across hospital-size categories.

### 5.3 Weighted VRS technical efficiency by hospital size group

Based on the results reported in Table 4, weighted technical efficiency under Variable Returns to Scale (VRS) ranged between 0.85 and 0.97 over the period 2019–2023. Over the full study period, overall bed-weighted VRS efficiency was 0.876 for small, 0.955 for medium-sized, and 0.960 for large hospitals. These values are systematically higher than the corresponding CRS efficiency scores, indicating that a substantial share of overall inefficiency is attributable to non-optimal operating scale rather than to pure technical or managerial inefficiency (Banker *et al.*, 1984; Coelli *et al.*, 2005).

**Table 4:** Weighted VRS technical efficiency by hospital size group

Year	Small (≤200)	Medium (201–400)	Large (≥401)
2019	0.896	0.947	0.970
2020	0.869	0.952	0.968
2021	0.856	0.953	0.936
2022	0.884	0.965	0.961
2023	0.878	0.958	0.967
2019–2023	0.876	0.955	0.960

**Note(s):** Weighted efficiency scores were calculated using hospital beds as weights. The 2019–2023 values represent the overall bed-weighted efficiency scores calculated from all hospital-year observations over the full study period

**Source(s):** ESY.net (2019–2023), Author’s own work

Medium-sized hospitals (201–400 beds) exhibit consistently high levels of pure technical efficiency, with an overall bed-weighed VRS efficiency of 0.955 for 2019–2023 and annual VRS scores remaining approximately between 0.95 and 0.97 throughout the study period. This finding suggests effective resource management and operational stability in hospitals of intermediate scale, largely independent of scale effects.

Large hospitals (≥401 beds) record the highest VRS efficiency scores, with an overall bed-weighted VRS efficiency of 0.960 for 2019–2023 and annual values reaching up to 0.97, reflecting strong pure technical performance. However, a noticeable decline is observed in 2021 (VRS = 0.936), which coincides with the peak of the COVID-19 pandemic and likely reflects extraordinary operating conditions and resource reallocation during that year. The subsequent recovery in 2022–2023 indicates a return towards more regular production conditions.

In contrast, small hospitals (≤200 beds) display systematically lower VRS efficiency scores, with an overall bed-weighted VRS efficiency of 0.876 for 2019–2023 and annual scores remaining below 0.90 throughout the five-year period. This pattern suggests comparatively weaker pure technical efficiency even after controlling for scale effects, potentially associated with administrative and organizational constraints.

Overall, the VRS results capture pure technical efficiency after accounting for scale effects, while the difference between VRS and CRS efficiency scores primarily reflects scale inefficiency. This reinforces the importance of jointly examining CRS and VRS models when assessing hospital performance (Banker *et al.*, 1984; Coelli *et al.*, 2005).

Using 2019 as the pre-pandemic reference point, the full-period VRS results show a modest decline for small and large hospitals, alongside an improvement for medium-sized hospitals. Overall bed-weighted VRS efficiency declined from 0.896 to 0.876 for small hospitals and from 0.970 to 0.960 for large hospitals, whereas it increased from 0.947 to 0.955 for medium-sized hospitals. This comparison therefore indicates that pure technical efficiency followed different patterns across hospital-size categories over the full study period.

### 5.4 Types of returns to scale (RTS)

Based on the results reported in Table 5, mean RTS scores are negative across all hospital-size groups and years, indicating an overall tendency toward decreasing returns to scale. This finding is consistent with the RTS classification results, as DRTS represents the predominant regime in most group-year observations.

**Table 5:** Types of returns to scale by hospital size group

Group	Year	Mean RTS score	IRTS (% of hospitals)	CRTS (% of hospitals)	DRTS (% of hospitals)
Small (≤200 beds)	2019	−0.034	35.19	3.70	61.11
	2020	−0.046	40.74	12.96	46.30
	2021	−0.010	35.19	14.81	50.00
	2022	−0.118	29.63	7.41	62.96
	2023	−0.030	29.09	18.18	52.73
Medium (201–400 beds)	2019	−0.112	8.0	12.0	80.0
	2020	−0.344	0.0	12.5	87.5
	2021	−0.137	3.85	42.30	53.85
	2022	−0.140	3.7	33.33	62.96
	2023	−0.234	0.0	61.54	38.46
Large (≥401 beds)	2019	−0.132	0.0	38.89	61.11
	2020	−0.209	0.0	45.0	55.0
	2021	−0.146	5.56	44.44	50.0
	2022	−0.122	5.88	35.29	58.82
	2023	−0.184	5.88	47.06	47.06

**Note(s):** Mean RTS score represents the arithmetic mean of the individual hospital-level RTS scores within each hospital-size group and year

**Source(s):** ESY.net (2019–2023), Author’s own work

Small and medium-sized hospitals exhibit particularly high proportions of DRTS, indicating that a substantial share operate above the Most Productive Scale Size (MPSS), where proportional increases in inputs result in less-than-proportional increases in outputs. Small hospitals nevertheless show comparatively higher proportions of IRTS, indicating that some operate below the MPSS.

Large hospitals display comparatively higher shares of CRTS, particularly toward the end of the study period, suggesting that a greater proportion operate close to the optimal scale. Overall, the combined evidence from mean RTS scores and the distribution of IRTS, CRTS, and DRTS provides a consistent indication of the prevailing scale characteristics across hospital-size groups.

### 5.5 Comparison with previous studies

Table 6 presents a comparison of hospital-size group performance between the present study and Fourlopoulou *et al.* (2025).

**Table 6:** Comparison of hospital-size group performance between the present DEA study (97–98 Greek public general hospitals; 97 hospitals in 2019 and 98 hospitals during 2020–2023) and Fourlopoulou *et al.* (2025) (109 Greek public hospitals, 2009–2021)

Hospital size group	Mean CRS TE	CRS rank	Mean VRS TE	VRS rank	CMgm cost productivity ranking (Fourlopoulou *et al.*, 2025)	Times ranked 1st (2009–2021, *n* = 13 years)
Small (≤200 beds)	0.835	3	0.877	3	3	1
Medium (201–400 beds)	0.920	2	0.955	2	1	9
Large (≥401 beds)	0.941	1	0.960	1	2	3

**Note(s):** CRS and VRS rankings are based on weighted DEA technical efficiency scores for 97–98 Greek public general hospitals (2019–2023). CMgm rankings are derived from the Multi Group Cost Malmquist Index reported by Fourlopoulou *et al.* (2025) for 109 Greek public hospitals (2009–2021) and represent cost productivity performance

**Source(s):** Author’s own work

Fourlopoulou *et al.* (2025) evaluated hospital performance using the Multi Group Cost Malmquist Index (CMgm), a measure of cost productivity, whereas the present study assessed technical efficiency using DEA-CRS and DEA-VRS models. Despite methodological differences, both studies indicate that hospital size is an important determinant of performance. Small hospitals demonstrated the weakest performance in both studies, while medium-sized and large hospitals demonstrated superior performance compared with small hospitals. However, Fourlopoulou *et al.* ranked medium-sized hospitals first overall, whereas the present study identified large hospitals as the most technically efficient group. Based on CMgm rankings, medium-sized hospitals ranked first in 9 of the 13 study years, followed by large hospitals (3 years) and small hospitals (1 year). These differences may reflect the fact that Fourlopoulou *et al.* evaluated cost productivity, whereas the present study focused on technical efficiency. Consequently, the most favorable hospital-size group may vary according to the performance dimension being assessed.

Table 7 summarizes the methodological and efficiency-related differences between the present study and Mitakos and Mpogiatzidis (2024).

**Table 7:** Methodological and efficiency comparison between Mitakos and Mpogiatzidis (2024) and the present study

Characteristic	Mitakos and Mpogiatzidis (2024)	Present study
Country	Greece	Greece
Sample	38 public hospitals	97 public general hospitals in 2019 and 98 public general hospitals during 2020–2023
Study period	2020	2019–2023
DEA orientation	Input-oriented	Input-oriented
CRS specification	CCR-I	CRS
VRS specification	BCC-I	VRS
Mean CRS efficiency	0.658	Small: 0.835; Medium: 0.920; Large: 0.941
Mean VRS efficiency	0.789	Small: 0.877; Medium: 0.955; Large: 0.960
Hospital-size analysis	Not performed	Small, Medium and Large hospitals analysed
Scale efficiency analysis	Not reported	Mean Scale Efficiency analysed by hospital size
Main efficiency pattern	Higher efficiency under VRS than CRS assumptions	Higher efficiency under VRS than CRS assumptions
		Efficiency scores increased consistently with hospital size (Large > Medium > Small) under both CRS and VRS models

**Note(s):** CCR-I and BCC-I correspond to CRS and VRS DEA specifications, respectively. Efficiency values reported by Mitakos and Mpogiatzidis (2024) refer to the overall hospital sample, whereas the present study reports efficiency by hospital-size group

**Source(s):** Author’s own work

Mitakos and Mpogiatzidis (2024) reported mean efficiency scores of 0.658 under CCR-I and 0.789 under BCC-I specifications for a sample of 38 Greek public hospitals during 2020. Similar to the present study, higher efficiency levels were observed under VRS assumptions than under CRS assumptions, suggesting the existence of scale inefficiencies within the Greek public hospital sector. Unlike Mitakos and Mpogiatzidis, who evaluated hospitals as a single group, the present study further examined efficiency by hospital size and scale efficiency patterns. This additional analysis revealed a consistent efficiency gradient across hospital-size groups, with larger hospitals exhibiting higher technical efficiency scores under both CRS and VRS assumptions.

## 6. Discussion and policy implications

### 6.1 General interpretation of results

When considered over the full 2019–2023 study period, the DEA analysis of Greek general hospitals indicates relatively high levels of pure technical efficiency under Variable Returns to Scale (VRS), particularly for medium-sized and large hospitals (approximately 0.96), while small hospitals exhibit slightly lower yet still satisfactory performance levels (around 0.88). In contrast, technical efficiency scores under Constant Returns to Scale (CRS) are systematically lower (approximately 0.84–0.94), suggesting that a substantial share of overall inefficiency is attributable to scale-related constraints rather than to purely technical or managerial shortcomings. The comparison with previous studies further supports the importance of hospital size as a determinant of hospital efficiency performance. Importantly, the comparison of the pre-pandemic year 2019 with overall performance during 2019–2023 reveals heterogeneous patterns across hospital-size categories. Small hospitals recorded lower overall CRS and VRS efficiency than in 2019, while their scale efficiency remained virtually unchanged. In contrast, medium-sized hospitals exhibited higher overall CRS, VRS, and scale efficiency, whereas large hospitals recorded somewhat lower overall CRS and VRS efficiency and only a marginal reduction in scale efficiency. These findings indicate that efficiency patterns over the full study period differed across hospital-size categories and cannot be interpreted solely in terms of a uniform COVID-19-related effect.

The majority of hospital units, especially within the small and medium-sized categories, operate under Decreasing Returns to Scale (DRTS), indicating operation beyond the Most Productive Scale Size (MPSS). This finding implies that further increases in inputs -such as staffing or operating expenditures-are not associated with proportional increases in outputs, a pattern consistent with the presence of organizational rigidities and limited efficiency of internal production flows.

Large hospitals consistently display higher technical efficiency scores and higher proportions of operation under Constant Returns to Scale (CRTS), particularly after 2020. This pattern suggests convergence toward the most productive scale of production and more effective adjustment of operating size, in line with findings from the international literature highlighting the role of economies of scale, improved internal organization, and higher levels of managerial and digital maturity (Worthington, 2004; Hollingsworth, 2008; Ozcan, 2014).

The divergence observed in 2021 in CRS efficiency scores-where medium-sized hospitals exhibit comparatively higher efficiency-coincides with the peak of the COVID-19 pandemic and is likely to reflect transitional operating conditions, resource reallocation, and emergency organizational adaptations rather than a permanent structural advantage of a specific size category (World Health Organization, 2021).

Overall, the results suggest that the Greek hospital system operates to a large extent in a technically efficient manner, while still exhibiting clear potential for optimization in terms of production scale and resource allocation. The findings support the need for targeted policies aimed at enhancing managerial flexibility, regional reorganization, and functional restructuring-particularly for smaller hospitals facing persistent scale-related constraints and underutilization of infrastructure. These interpretations should be considered in light of the well-known limitations of DEA, especially the absence of adjustment for case-mix and qualitative dimensions of service provision.

### 6.2 Integrated discussion: efficiency, human resources, and policy implications

The analysis of Proportionate Movements, Slack Movements, and Dual Prices within the CRS-based Data Envelopment Analysis (DEA) framework for Greek general hospitals over the period 2019–2023 reveals substantial differences in resource management and workforce utilization across hospital size groups.

#### 6.2.1 Efficiency and resource utilization by hospital size

Small hospitals (≤200 beds) exhibit an overall bed-weighted CRS technical efficiency score of 0.835 over the full 2019–2023 study period, with estimated potential reductions in inputs (proportionate movements) ranging from 15% to 18% and additional non-radial adjustment margins (slacks) of approximately 15%–20%, primarily associated with operating (administrative) and pharmaceutical expenditures. The lower efficiency observed in this group is closely linked to the geographical and demographic characteristics of the areas served, as well as persistently low occupancy rates, which constrain economies of scale and increase unit operating costs.

Medium-sized hospitals (201–400 beds) record an overall bed-weighted CRS technical efficiency score of 0.921 over the full 2019–2023 study period, with proportionate movements of 7%–10% and slack adjustments of 10%–12%. These results indicate a more coherent and rational use of resources, although organizational inefficiencies remain. These findings are consistent with Fourlopoulou *et al.* (2025), who identified medium-sized hospitals as the best-performing category in terms of cost productivity among Greek public hospitals. Although productivity and technical efficiency represent different performance dimensions, the convergence of findings across the two studies suggests that medium-sized hospitals may achieve a more balanced utilisation of resources and organisational capacity than either smaller or larger units. In contrast, large hospitals (≥401 beds) demonstrate the highest overall bed-weighted CRS technical efficiency, with a score of 0.941 over the full 2019–2023 study period combined with limited adjustment potential (proportionate movements of 5%–7% and slacks below 5%), reflecting more mature administrative structures and more effective integration of existing inputs. This finding confirms the pivotal role of operating scale as a determinant of technical efficiency in the public hospital sector, consistent with evidence from the international literature (Ozcan, 2014; Hollingsworth, 2008).

Dual prices (shadow prices) remain low (0.00–0.015) for most input variables, indicating that simple increases in resource allocation would not lead to substantial efficiency gains. By contrast, positive shadow prices associated with the training output of resident physicians (0.02–0.03) suggest that investment in medical training and the strengthening of residency programs could improve efficiency by up to 3%. Taken together, slack movements were interpreted primarily as recurring system-level indicators of operational inefficiency rather than isolated deviations at the individual hospital level.

Overall, the findings point to imbalances in the allocation of expenditures and nursing staff, with a significant share of resources remaining underutilized or inefficiently deployed. These findings may partly reflect workforce shortages and uneven staffing distribution patterns across the Greek public hospital system (Athanasakis *et al.*, 2022). This condition constrains technical efficiency and may adversely affect service quality. The results highlight the need for rationalization of operating expenditures and reallocation of human resources in order to enhance technical adequacy and organizational effectiveness within the Greek public hospital system.

#### 6.2.2 Temporal trends and COVID-19

Beyond the year-specific variations observed during the pandemic, comparison of the pre-pandemic 2019 benchmark with overall performance across 2019–2023 provides a broader perspective on the heterogeneous efficiency patterns observed across hospital-size categories. This comparison indicates that the full-period efficiency pattern was not uniform across hospital-size categories: medium-sized hospitals improved relative to the pre-pandemic benchmark, whereas small and large hospitals showed lower overall CRS and VRS efficiency, with comparatively limited changes in scale efficiency. Nevertheless the COVID-19 pandemic constituted a critical turning point for the Greek health system, with measurable effects on hospital efficiency. In 2020–2021, variations in CRS efficiency scores were observed across hospital-size categories, reflecting heterogeneous performance patterns during the pandemic period and the temporary reallocation of human and financial resources toward crisis management. From 2022 to 2023, efficiency patterns were mixed across hospital-size categories and DEA measures, although scale efficiency improved across all three size categories.

These patterns should be considered in the context of broader improvements in management practices and digital support within the health sector, as documented in international reports (OECD, 2021; Ministry of Digital Governance, 2020; OECD/European Observatory on Health Systems and Policies, 2023). These sources highlight the acceleration of digital transformation-primarily through the strengthening of information systems and electronic services-as a key factor supporting administrative continuity and operational resilience. Efficiency performance remained comparatively stronger among medium-sized and large hospitals, while smaller hospitals continue to face constraints related to limited resources and technological capacity. These differences may reflect geographical and administrative influences on hospital productivity, consistent with recent findings in Greece (Fourlopoulou *et al.*, 2025). Such interventions may also contribute to reducing regional inequalities in healthcare access, particularly in geographically remote areas.

Within this context, the findings underscore the need for organisational restructuring and targeted strengthening of small hospitals, particularly in island, mountainous, and other geographically constrained areas, through the effective use of technology and the upgrading of information systems. Such interventions could enhance accessibility, operational resilience, and overall technical efficiency within the public health sector. These results are aligned with international evidence pointing to a post-pandemic recovery in operational efficiency and emphasize the importance of sustained investments in technology and organizational flexibility for the long-term improvement of hospital performance (OECD and European Commission, 2024).

#### 6.2.3 Policy and administrative implications

The results of the DEA analysis for the period 2019–2023 highlight distinct policy and administrative priorities depending on hospitals' scale characteristics and returns-to-scale regimes. For hospitals operating under Decreasing Returns to Scale (DRTS), which represent the predominant RTS regime in most group-year observations as indicated by the empirical findings, service reorganization and the strengthening of internal control mechanisms are recommended to reduce functional overlaps and achieve more efficient use of system resources (OECD, 2021). These considerations may be particularly relevant for large hospitals in urban centers, given the concentration of health care services and facilities in urban settings (OECD/European Observatory on Health Systems and Policies, 2021). In hospitals facing high clinical and administrative burdens, the expansion of extended-hour services and the consolidation of similar departments may enhance efficiency without generating additional fiscal costs (Kounetas and Papathanassopoulos, 2013).

In contrast, hospitals operating under Increasing Returns to Scale (IRTS)−observed predominantly among small hospitals in the present analysis*,* as reflected in the RTS distribution−require targeted investments in infrastructure and human resources to strengthen productive capacity and reduce spatial and technological disparities (WHO, 2022; OECD/European Observatory on Health Systems and Policies, 2023; Hellenic Ministry of Health, 2023). Particular emphasis should be placed on the renewal and reinforcement of the healthcare workforce, given the demographic ageing of personnel and the increasing outflow of health professionals in the Greek public health system, which constrain operational flexibility and the ability to deliver high-quality services (OECD and European Commission, 2024; WHO, 2022). This is consistent with recent evidence emphasizing workforce imbalances and planning challenges within the Greek public health system (Athanasakis *et al.*, 2022).

For hospitals operating under Constant Returns to Scale (CRTS), further progress should be primarily associated with quality improvement and innovation. This includes the implementation of patient safety and quality protocols (Donabedian, 1988; OECD, 2021), digital transformation initiatives (electronic health records, telemedicine, data analytics), and the adoption of modern management practices such as performance monitoring through Key Performance Indicators (KPIs) and benchmarking, aimed at continuous improvement in quality, accountability, and service efficiency (European Commission, 2022).

Within the Greek context−where the uneven distribution of personnel and infrastructure remains a persistent challenge−targeted support for smaller and regionally disadvantaged hospitals, systematic workforce renewal and training, and the effective use of digital technologies can significantly contribute to improving efficiency and the long-term sustainability of the public hospital system (OECD, 2021; WHO, 2022; OECD/European Observatory on Health Systems and Policies, 2023). This is consistent with recent evidence highlighting the importance of reducing regional disparities and strengthening health-system resilience in Greece (Fourlopoulou *et al.*, 2025).

As part of recent reform initiatives, the development and pilot implementation of performance indicators (KPIs) by the National Authority for Quality Assurance in Health Care (ODIPY) has been underway since 2022 within the framework of the *Greece 2.0* program (Hellenic Ministry of Finance, 2022; ODIPY, 2023). This initiative is expected to substantially strengthen systematic efficiency monitoring and enable more accurate benchmarking across hospitals in the future.

Although the present study does not incorporate these indicators, their full implementation and utilization may improve data availability and quality for future DEA analyses, while also enhancing transparency and accountability mechanisms within the National Health System. Overall, the findings support a shift from uniform, horizontal policy measures toward differentiated interventions, tailored to hospitals' operating scale and efficiency profiles.

### 6.3 Key takeaways/synthesis of findings

The discussion of the results yields five key findings with direct implications for the organization and governance of the National Health System.

1. First, large hospitals act as structural benchmarks for the system, exhibiting consistently high efficiency levels and greater organizational maturity.
2. Second, medium-sized hospitals retain substantial potential for further efficiency gains, primarily through targeted managerial support and more rational resource reallocation.
3. Third, small hospitals−particularly those located in island, remote, and sparsely populated areas−require organizational restructuring or the development of network-based collaborations with larger units to ensure long-term operational sustainability.
4. Fourth, integrating DEA-based efficiency assessment into the Ministry of Health's performance indicator framework (KPIs) could enhance objectivity and transparency in resource distribution and hospital performance evaluation.
5. Finally, the incorporation of returns-to-scale (RTS) evidence into policy decision-making provides empirically grounded guidance for expansion, restructuring, or potential consolidation of hospital units, with the aim of strengthening long-term sustainability within the public hospital system.

## 7. Limitations and directions for future research

### 7.1 Limitations

Although the findings of this study provide a coherent overview of efficiency patterns among Greek public hospitals, several limitations should be acknowledged.

First, the analysis relies exclusively on administrative input-output data and does not incorporate indicators of quality of care, case severity, or clinical outcomes. As a result, measured technical efficiency does not necessarily correspond to higher quality of service provision.

Second, intertemporal comparisons are influenced by the exceptional conditions associated with the COVID-19 pandemic, which temporarily altered demand patterns, staffing levels, and organizational arrangements across hospitals. Within this context, slack analysis was used primarily to identify recurring operational inefficiency patterns across the hospital system rather than isolated unit-level deviations.

Third, differences observed between small, medium-sized, and large hospitals should be interpreted with caution, as spatial, demographic, and organizational characteristics−such as geographic isolation or population density−are not explicitly captured in the model but may substantially affect efficiency outcomes.

Finally, the study focuses on technical efficiency rather than economic or clinical efficiency, indicating the need for complementary analytical approaches before drawing binding policy conclusions.

### 7.2 Directions for future research

Future research could extend the present analysis through the application of bootstrapped DEA techniques to assess the statistical robustness of efficiency estimates. The adoption of two-stage DEA models incorporating environmental and organizational variables would allow for the identification of key determinants of hospital efficiency.

The inclusion of quality indicators, clinical outcomes, and case-mix adjustments (e.g. DRGs) would further enhance the comparability and interpretability of results. Moreover, combining DEA with cost-efficiency models or stochastic frontier analysis (SFA), as well as systematically examining organizational and managerial characteristics−such as leadership quality, governance structures, and digital maturity−could provide a more comprehensive framework for evaluating hospital performance and efficiency differentials across units.

## 8. Conclusions

This study assessed the technical efficiency of Greek general hospitals over the period 2019–2023 using Data Envelopment Analysis under Constant Returns to Scale (CRS) and Variable Returns to Scale (VRS). The results indicate high levels of pure technical efficiency under VRS for medium-sized and large hospitals (≈0.95–0.97), while smaller hospitals exhibit lower efficiency scores (≈0.88), suggesting that a substantial share of observed inefficiency is attributable to non-optimal operating scale rather than technical constraints (Charnes *et al.*, 1978; Banker *et al.*, 1984; Coelli *et al.*, 2005).

Overall technical efficiency under CRS averaged around 0.90, with large hospitals operating closer to the Most Productive Scale Size (MPSS), reflecting greater organizational maturity, stronger managerial capacity, and more effective resource utilization (Ozcan, 2014; Worthington, 2004). Scale efficiency (SE = TE_CRS/TE_VRS) remained relatively high throughout the study period, with large hospitals exhibiting the highest overall mean scale efficiency (0.981) followed by medium-sized (0.963) and small hospitals (0.946). Between 2022 and 2023, scale efficiency increased across all hospital-size categories, a pattern consistent with broader health-system adaption during the post-pandemic period (OECD and European Commission, 2024).

Nevertheless, Decreasing Returns to Scale (DRTS), represent the predominant RTS regime in most hospital-size group and year observations, indicating persistent scale-related inefficiencies and the need for improved resource allocation and management. In contrast, a subset of smaller hospitals operates under Increasing Returns to Scale (IRTS), highlighting potential opportunities for efficiency gains through targeted investments in infrastructure and human resources.

The intertemporal evolution of efficiency indicators indicates a general stabilization of hospital performance during 2022–2023 following the pandemic-related disruptions observed in the preceding years. These findings provide clear policy-relevant insights:

1. the need for targeted administrative restructuring and cooperative arrangements among smaller hospitals,
2. the preservation of optimal operating scale in large hospitals with an emphasis on quality and innovation, and
3. the systematic integration of DEA into the evaluation, benchmarking, and evidence-based resource allocation framework of the National Health System.

Overall, the study demonstrates the value of DEA as a robust tool for assessing hospital efficiency and provides evidence-based insights for improving resource allocation and operational performance within the Greek public healthcare system.
